# 589. Late Onset Invasive Group B Streptococcal Disease Outbreak in a Neonatal Intensive Care Unit Identified Through Whole Genome Sequencing — Connecticut, 2020–2024

**DOI:** 10.1093/ofid/ofae631.184

**Published:** 2025-01-29

**Authors:** Matthew C Lambert, Sydney Jones, Kelly Mueller, Meghan Maloney, Neranjan Perera, Kutluhan Incekara, Susan Petit, Veena Ramachandran, Marissa Grossman, Amy Valderrama, Sopio Chochua, Lesley McGee, Benjamin Metcalf, Stephanie Schrag, Lynn Sosa

**Affiliations:** Centers for Disease Control, Hartford, CT; Centers for Disease Control, Hartford, CT; Connecticut Department of Public Health, Hartford, Connecticut; Connecticut Department of Public Health, Hartford, Connecticut; Connecticut Department of Public Health Laboratory, Rocky Hill, Connecticut; Connecticut Department of Public Health Laboratory, Rocky Hill, Connecticut; Connecticut Department of Public Health, Hartford, Connecticut; Centers for Disease Control and Prevention, Atlanta, GA; Centers for Disease Control and Prevention, Atlanta, GA; Centers for Disease Control, Hartford, CT; CDC, Atlanta, Georgia; Centers for Disease Control and Prevention, Atlanta, GA; Centers for Disease Control, Hartford, CT; CDC, Atlanta, Georgia; Connecticut Department of Public Health, Hartford, Connecticut

## Abstract

**Background:**

Group B *Streptococcus* (GBS) is a major cause of neonatal sepsis. Outbreaks of GBS late-onset disease (LOD), defined as infection during the first 7–89 days of life, are uncommon. In September 2023, 4 GBS LOD cases were reported by a neonatal intensive care unit (NICU) to the Connecticut Department of Public Health (DPH). We investigated to characterize the outbreak and guide response.

**Methods:**

We identified patients with invasive (i.e., GBS isolated from a sterile site) LOD hospitalized in the NICU through review of data from DPH’s Active Bacterial Core Surveillance Program during January 2019–April 2024. We distinguished outbreak cases through whole-genome sequencing (WGS) of associated bacterial isolates. Single nucleotide polymorphism (SNP) analysis with a cutoff of ≤12 SNPs defined a case as outbreak-related. We abstracted medical records to collect case data. We performed 3 on-site assessments of infection prevention and control (IPC) practices and policies. To monitor response effectiveness, we performed weekly GBS colonization screening (aural, oropharyngeal, rectal sites) of infants admitted to the NICU during December 2023–April 2024. We performed WGS on GBS isolates associated with each colonization.

**Results:**

We identified 12 outbreak-related cases during January 2020–April 2024 based on WGS. Median case interval, time between illness onset, was 85 days but ranged from three days to 458 days; 3 outbreak-patients died. Chart abstraction and on-site assessments indicated the milk room as an area for potential transmission. Variability was noted in adherence to appropriate hand hygiene, personal protective equipment use, and environmental cleaning throughout the NICU. Screening identified 7 colonized infants; GBS isolates from 3 infants matched the outbreak, and 1 colonized infant developed LOD. We recommended addressing IPC gaps and continuing GBS colonization screening to monitor for ongoing transmission.

**Conclusion:**

This investigation highlights the integration of WGS with classic epidemiology to distinguish outbreak-related cases with long intervals between identification. In LOD GBS outbreaks where IPC gaps appear to contribute to transmission, colonization screening and WGS can evaluate effectiveness of implementation of protocols to address IPC gaps.

**Disclosures:**

**Meghan Maloney, MPH**, Pfizer Global R&D: Former employee, separated in 2009. I do have a retirement entitlement which I do not actively manage/no stock options. PGRD offers periodic buy outs

